# Stress-dependent activation of the *Listeria monocytogenes* virulence program ensures bacterial resilience during infection

**DOI:** 10.1128/mbio.00719-25

**Published:** 2025-04-30

**Authors:** Mariya Lobanovska, Ying Feng, Jonathan Zhang, Allison H. Williams, Daniel A. Portnoy

**Affiliations:** 1 Department of Molecular and Cell Biology, University of California, Berkeley 196203, Berkeley, California, USA; 2 Department of Cellular and Molecular Pharmacology, University of California, San Francisco 371259, San Francisco, California, USA; 3 Chan Zuckerberg Biohub 578083, San Francisco, California, USA; 4 Department of Plant and Microbial Biology, University of California, Berkeley 118549, Berkeley, California, USA; University of Illinois Chicago, Chicago, Illinois, USA

**Keywords:** intracellular bacteria, pathogenesis, stress adaptation, sigma factors, virulence regulation, macrophages

## Abstract

*Listeria monocytogenes (Lm*) is a Gram-positive, facultative intracellular pathogen that uses both a housekeeping (P1) and stress-activated (Sigma B-dependent) promoter (P2) to express the master virulence regulator PrfA. The Sigma B regulon contains over 300 genes known to respond to different stressors. However, the role of Sigma B in the regulation of *prfA* during the infection remains uncertain. To define pathways that lead to Sigma B-dependent *prfA* activation, we performed a genetic screen in L2 fibroblasts using ΔP1 *Lm* that only has the Sigma B-dependent promoter directly upstream of *prfA*. The screen identified transposon insertions in a large bacterial sensory organelle known as the stressosome. The absence of functional stressosome components resulted in heterogeneity within bacterial populations, with some bacteria behaving like wild type, while other members of the population exhibited defects in either vacuolar escape and/or cell-to-cell spread. We show that the heterogeneity of the stressosome mutants cannot be rescued by constitutive activation of PrfA. These data defined the importance of the stressosome in controlling bacterial homogeneity and characterized the function of the stressosome in robust virulence activation during infection. ΔP1 *Lm* model provides new opportunities to identify host-specific signals necessary for stressosome-dependent signaling during *Listeria* pathogenesis.

**IMPORTANCE:**

Microbial pathogens must adapt to varying levels of stress to survive. This study uncovered a link between stress sensing and activation of the virulence program in a facultative intracellular pathogen, *Listeria monocytogenes*. We show that host-imposed stress is sensed by the signaling machinery known as the stressosome to ensure robust and resilient virulence responses *in vivo*. Stressosome-dependent activation of the master virulence regulator PrfA was necessary to maintain *L. monocytogenes* homogeneity within the bacteria population during the transition between early and late stages of intracellular infection. This work also provides a model to further characterize how specific stress stimuli affect bacterial survival within the host*,* which is critical for our understanding of bacterial pathogenesis.

## INTRODUCTION

*Listeria monocytogenes* (*Lm*) is a Gram-positive facultative intracellular bacterium capable of causing serious infections in a wide range of mammalian hosts (1). The *Lm* intracellular lifecycle begins upon entering a phagosome. *Lm* then escapes the phagosome and enters the host cytosol, where it replicates (1, 2). *Lm* mediates the polymerization of host cell actin to propel through the cytosol, move from one cell to another, and cause infection in the neighboring cell where its intracellular life cycle repeats (3). Each step in the intracellular life cycle is carefully orchestrated by virulence factors, the expression of which is mediated by the essential virulence regulator PrfA (2).

**Fig 1:**
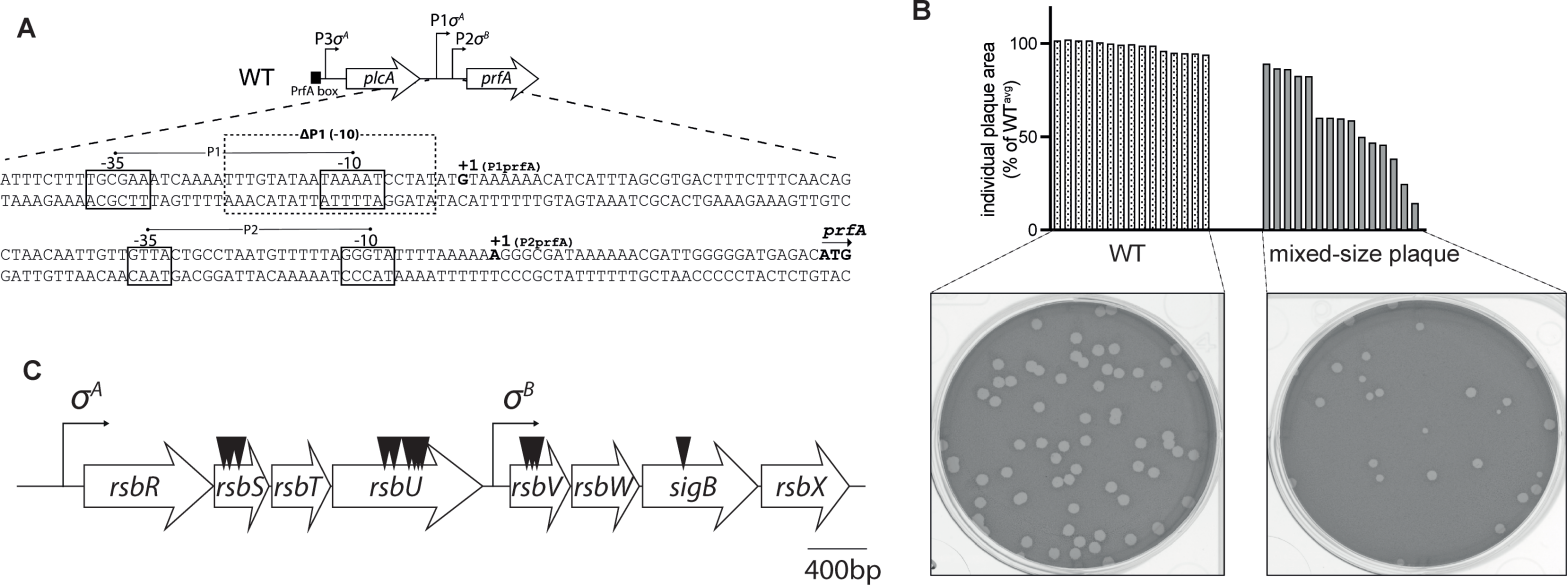
Mixed-size plaque phenotype in a ΔP1 background. (**A**) Schematic of the ΔP1 *Lm* strain used in the genetic screen. The ΔP1 strain contains a deletion of the −10 sequence of the Sigma A-dependent P1 promoter. Physical map of *prfA* promoter region of *Lm* is adapted from Freitag and Portnoy (8). (**B**) Mixed-size plaque formation. Transposon mutants identified in the screen formed plaques of mixed sizes. The bar graph depicts plaque size morphology of a representative transposon mutant compared to WT plaques. (**C**) The stressosome locus. Eight-gene operon encoding different components of the stressosome signaling pathway contains a distal Sigma A-dependent promoter and Sigma B-dependent promoter positioned within the operon. Transposon insertion sites identified in the screen are shown by black arrowheads.

PrfA is regulated at multiple levels. At the transcriptional level, there are three promoters upstream of *prfA*: P1 and P2 are directly upstream of *prfA*, each generating a monocistronic transcript, while P3 is upstream of *plcA*, a PrfA-dependent gene encoding PlcA, that generates a biscistronic transcript *plcA-prfA* (Fig. 1A). The *prfA* 5′UTR contains a thermosensor, which is subject to further regulation *via* SsrA binding (4, 5). The PrfA protein is allosterically activated by binding glutathione (6), which is synthesized by a bacterial glutathione synthase (GshF), encoded by *gshF (7*). Activated PrfA protein forms a dimer that binds to a palindromic PrfA-binding DNA sequence known as the PrfA box to enhance the transcription of *prfA* and PrfA-dependent genes. There is a PrfA box upstream of *plcA* resulting in a positive autoregulatory loop (8).

The three promoters upstream of *prfA* are regulated by different sigma factors. P1 and P3 are regulated by the housekeeping Sigma A factor, whereas P2 is regulated by Sigma B (9, 10). Sigma B is one of the four alternative sigma factors found in *Lm* and is thought to be the main stress response regulator. Sigma B regulates the expression of more than 300 genes associated with different stress signals (11–13). Although regulation of *prfA* has been extensively studied, the role of stress-dependent regulation of *prfA* at the Sigma B-dependent promoter during different stages of infection has not been clearly defined. Previous studies that examined the contribution of different *prfA* promoters identified P3 as the principal promoter driving *plcA-prfA* transcription during infection (14). P1 and P2 *prfA* promoters were considered redundant since the virulence of *Lm* was unaffected when either P1 or P2 was absent (8). In this study, we focused on understanding how *prfA* is regulated at the P2 Sigma B-dependent promoter. Using a *Lm* strain lacking the P1 promoter and therefore only having the Sigma B-dependent promoter directly upstream of *prfA,* we show that a large multicomponent bacterial stress-sensing machinery known as the stressosome is involved in regulating *prfA*. The stressosome responds to stress by triggering a partner-switching cascade resulting in Sigma B-dependent regulon activation (15). We show that the *Lm* stressosome can function *in vivo* and provide a model to dissect further bacterial stress sensing during infection.

## RESULTS

### Genetic screen to identify *Lm* genes necessary to activate the Sigma B-dependent *prfA* promoter

To identify regulators of *prfA* that required the Sigma B-dependent promoter, we used a strain with a deletion of the ΔP1 −10 box within the P1 Sigma A-dependent *prfA* promoter (8). The ΔP1 strain therefore only had the P2 Sigma B-dependent promoter directly upstream of *prfA* in addition to the P3 promoter positioned upstream of *plcA-prfA* (Fig. 1A). Removing the P1 promoter allowed us to reveal Sigma B-dependent regulators of *prfA,* as full activity of the P2 promoter is essential for *Lm* virulence when P1 is absent (8). We constructed a transposon library in a ΔP1 background and screened approximately 40,000 colonies in L2 fibroblasts to identify insertion mutants that had a visible plaque defect. We identified 15 small plaque mutants, and upon plaque purification, found nine mutants that displayed an unusual phenotype that manifested as plaques with heterogeneous size, which we call the mixed-size plaque phenotype (Fig. 1B). Genes identified in the screen (Table 1) included *sigB* and three genes that encode components of a stress-sensing machinery known as the stressosome, including *rsbS*, *rsbU*, and *rsbV*. Additional genes identified include components of the cytochrome D ubiquinol oxidase *cydA* and *cydD*, *lmo2456* encoding phosphoglycerate mutase, and two genes that have previously been implicated in *Lm* virulence, including *lmo1652* and *lmo1354* encoding a putative ABC transporter and aminopeptidase, respectively (16, 17). The majority of the mutants identified in the screen were located within an eight-gene operon encoding the stressosome (Fig. 1C), and we focused our subsequent analysis on these mutants.

**TABLE 1:** Genes identified by transposon mutations that cause a mixed-size plaque phenotype in a ΔP1 background

Gene name	LMRG/lmo number	# of insertions	Annotation
*rsbS*	02314/0890	3	Core stressosome component
*rsbU*	02316/0892	6	Stressosome signaling serine phosphatase
*rsbV*	02317/0893	3	Anti-anti-sigma factor (antagonist of RsbW)
*sigB*	02319/0895	1	Sigma factor B
*cydA*	01978/2718	1	Cytochrome D ubiquinol oxidase subunit I
*cydD*	01980/2715	1	ABC transporter ATP-binding protein
*pgm*	00712/2456	1	Phosphoglyceromutase
	00804/1354	4	Aminopeptidase P
	01315/1652	1	ABC transporter

### Mutants in stressosome components have virulence defects

**Fig 2:**
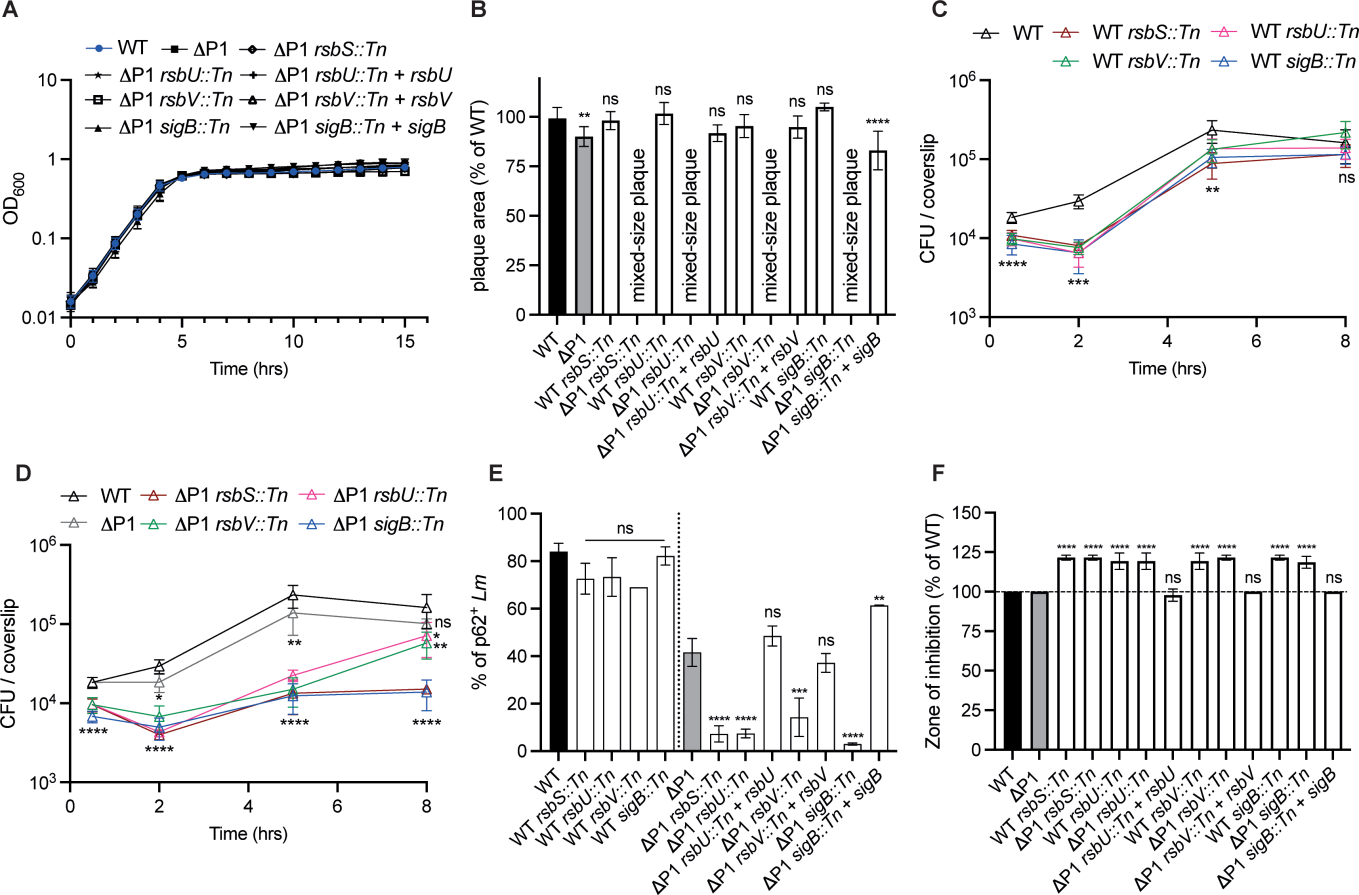
*In vitro* characterization of stressosome mutants. (**A**) Growth of the indicated strains at 37°C in BHI broth. Overnight bacterial cultures were normalized to an OD_600_ = 0.03 in fresh BHI and grown at 37°C for 15 h with agitation. The graph represents pooled data from three independent biological repeats. No statistical difference was detected between the strains. (**B**) Plaque area, presented as a percentage of WT, of indicated strains was measured following a 3-day infection of L2 cells. Data represent the mean of three independent experiments. (**C and D**) Growth curves in bone marrow-derived macrophages pre-treated with PAM3CK and infected with indicated strains at MOI of 0.25. Gentamicin (50 µg/mL) was added 1 h post-infection to kill extracellular bacteria. CFUs were counted at indicated time points, and the experiments were performed three times. (**E**) Quantification of cytosolic *Lm* expressed as a percentage of p62^+^ *Lm*^+^ bacteria. BMMs were infected with the indicated strains for 1.5 h. Cells were washed, fixed, and stained with anti-*Listeria* and anti-p62 antibodies. The percentage of *Lm* that escapes the phagosome and colocalizes with p62 is shown. At least 70 bacteria were analyzed per strain, and the experiment was performed two times. (**F**) Indicated strains were tested for sensitivity to the thiol-specific oxidant diamide by a disk diffusion assay. Zone of inhibition reflects the diameter presented as a percentage of WT. Data represent the mean of three independent experiments. For all experiments: Error bars indicate standard deviation. One-way ANOVA was used for multiple comparisons with WT (**A–D, F**) or WT and ΔP1 (**E**). ns, not significant, *P < 0.05; **P < 0.01; ***P < 0.001; ****P < 0.0001.

The stressosome is a multicomponent signaling machine that responds to environmental stimuli by activating Sigma B (Fig. S1) (18, 19). To investigate the role of the stressosome activity in virulence, we first examined the growth of the mutants under standard laboratory conditions in brain heart infusion (BHI) media at 37°C. The stressosome mutants displayed homogeneous colony morphology on BHI agar and showed a similar growth pattern to the wild-type (WT) and ΔP1 strains, suggesting that the mixed-size plaque observed in the tissue culture model is not due to the growth difference between the mutants (Fig. 2A; Fig. S2A). Next, we verified that the observed mixed-size plaque phenotype was associated with a ΔP1 background. Transposon insertions in the stressosome locus were transduced into WT and ΔP1 backgrounds, and the mutants were examined in L2 cells (Fig. 2B). The ΔP1 background strain had a minor plaque defect compared to WT, but the plaques were homogeneous in size. However, all of the mutants with insertions in genes encoding stressosome components exhibited the mixed-size plaque phenotype in a ΔP1 background. The mixed-size plaque phenotypes in ΔP1 *rsbU::Tn* and ΔP1 *rsbV::Tn* were fully rescued by complementing the strains with the corresponding genes under the control of the constitutive P*_hyper_* promoter. Complementation of ΔP1 *sigB::Tn* with P*_hyper_-sigB* resulted in a minor virulence defect likely because the overexpression of *sigB* has been suggested to be toxic for *Lm* (20). We were unable to complement ΔP1 *rsbS::Tn* with P*_hyper_-rsbS,* implying that constitutive expression of *rsbS* is detrimental to *Lm* (Fig. S3A).

**Fig 3:**
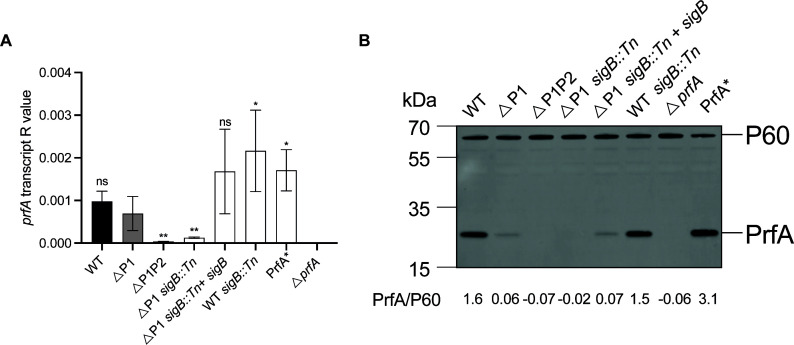
ΔP1 stressosome mutants have decreased PrfA expression. Indicated strains were grown to mid-log in iLSM + TCEP at 37°C, and samples were collected for RNA and Western blot analysis. (**A**) *prfA* expression was measured using qRT-PCR and normalized to 16S rRNA. One-way ANOVA was used to calculate for multiple comparisons with ΔP1. ns, not significant, *P < 0.05; **P < 0.01. (**B**) PrfA was detected using anti-PrfA antibody, and P60 expression was used as a control. The data represent three independent biological repeats. A ratio of PrfA/P60 was quantified using integrated density values.

To further define the virulence defects of the mixed-size plaque mutants, we switched to using bone marrow-derived mouse macrophages (BMMs), since macrophages are a primary cell infected by *Lm in vivo* (21). BMMs were treated with the TLR-agonist PAM3CK4 overnight prior to infection to mimic the stimulated state of macrophages during infection *in vivo*. Transposon mutants within the stressosome operon in a WT background displayed a defect early in infection (0–2 h). However, upon entry into the cytosol (2–8 h), the growth rate of the mutants was comparable to WT *Lm* (Fig. 2C). The growth of a ΔP1 strain had a minor defect at 2 and 5 h compared to WT, consistent with the slight ΔP1 plaque defect (Fig. 2D). Stressosome mutants in a ΔP1 background exhibited a defect early during infection similarly to WT stressosome mutants, suggesting that disruption of stressosome signaling impaired phagosomal escape of *Lm* irrespective of the background strain. Interestingly, stressosome mutants in a ΔP1 background were also attenuated during growth in macrophages at later times (5 and 8 h) (Fig. 2D). The defect seen in ΔP1 mutants was restored following complementation (Fig. S2B). Together, these data implicated a role for stressosome-dependent activation of the *prfA* P2 promoter throughout the *Lm* intracellular life cycle in a ΔP1 background.

To confirm the role of the stressosome mutants early during infection, we measured the ability of the mutants to escape from phagosomes (Fig. 2E). Following 90 min of infection, BMMs were stained for *Lm* and the autophagy receptor p62, which colocalizes with *Lm* that enters host cytosol (22). By measuring the percentage of *Lm*^+^ p62^+^ population, we can evaluate the ability of *Lm* to escape the phagosome. We observed that approximately 80% of WT bacteria and 40% of ΔP1 bacteria were p62 positive. Transposon insertions in the stressosome locus in a ΔP1 background were less than 20% p62 positive, confirming the role of P2 in *Lm* phagosomal escape. In the complemented strains, the defect was comparable to the ΔP1 background. The stressosome mutants in the WT background were able to escape the phagosome like WT, but these mutants showed an early growth defect in BMMs (Fig. 2E).

Since *Lm* harboring stressosome mutations exhibited a defect in phagosomal escape, we next investigated whether these mutants were sensitive to redox stress that might be encountered in the phagosome. We tested two redox stress stimuli, including hydrogen peroxide (H_2_O_2_) that imposes oxidative stress and diamide, which causes disulfide stress mimicking vacuolar conditions (23, 24). The sensitivity of the stressosome mutants in WT and ΔP1 backgrounds was comparable to WT in response to H_2_O_2_ using a disc diffusion assay (Fig. S2C). However, WT and ΔP1 stressosome mutants showed increased sensitivity to diamide (Fig. 2F), indicating that the stressosome can respond to disulfide stress that may be encountered during infection.

### Disruption of the stressosome signaling results in lower PrfA levels

Previous reports suggested that Sigma B directly interacts with the P2 promoter, and deletion of the P2 promoter leads to lower *prfA* levels (8, 25, 26). We therefore hypothesized that stressosome mutants in a ΔP1 background would similarly have lower PrfA levels. To test this, we measured PrfA transcript and protein levels in ΔP1 *sigB::Tn* grown in defined *Listeria* specific media iLSM supplemented with a reducing agent (tris(2-carboxyethyl)phosphine TCEP) that recapitulates *in vivo* environment (27). *prfA* transcripts were barely detectable in ΔP1 *sigB::Tn* compared to WT and ΔP1, consistent with *prfA* expression levels in the ΔP1P2 strain (Fig. 3A). Similarly, no PrfA protein was detected in a ΔP1 *sigB::Tn* which phenocopied ΔP1P2. This confirmed the direct effect of the disrupted stressosome signaling on PrfA expression in a ΔP1 background.

### P1 and P2 *prfA* promoter elements are required in the absence of glutathione synthesis

**Fig 4:**
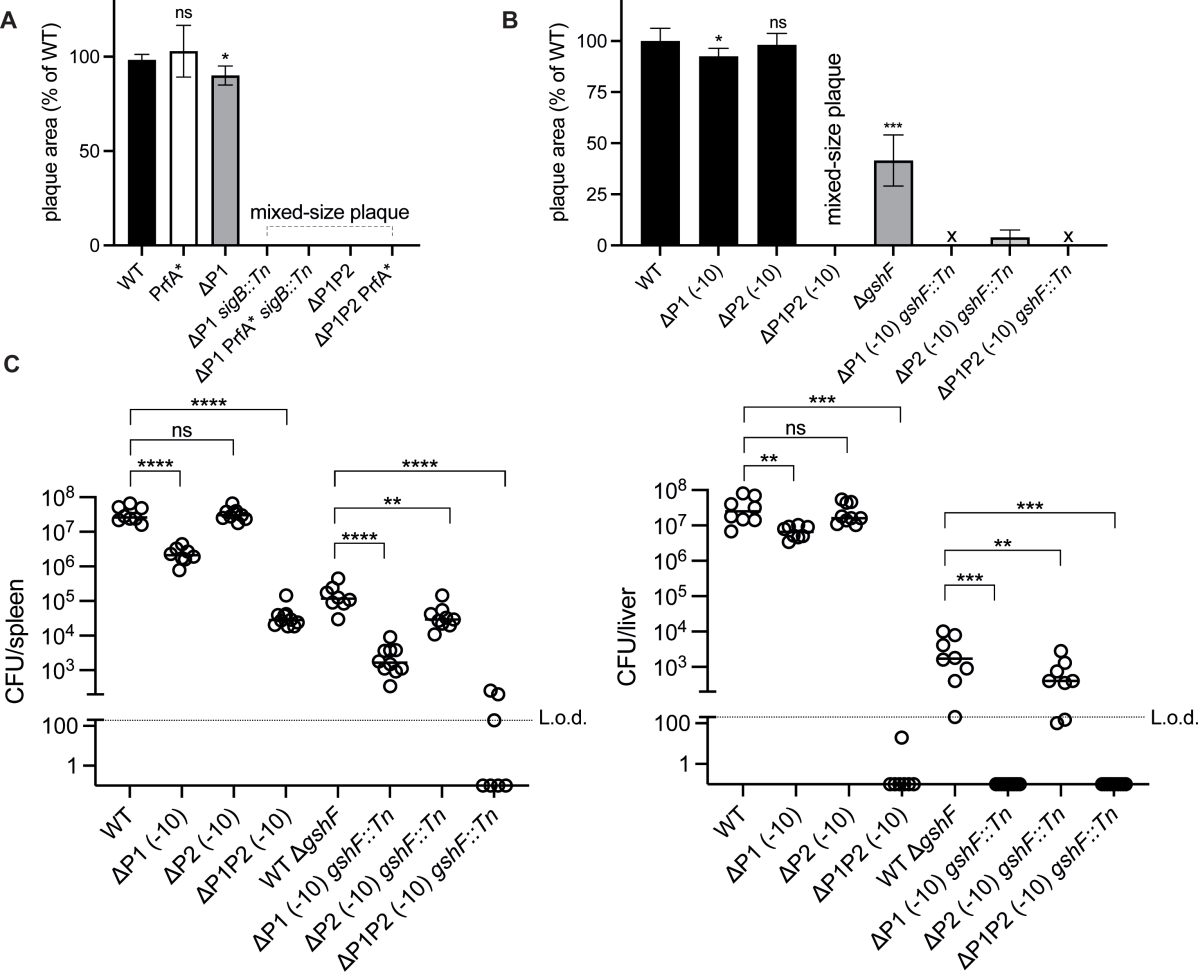
Role of PrfA activation in the virulence of *prfA* promoter mutants *in vivo*. (**A, B**) Plaque formation of indicated strains measured in the L2 plaque assay. Plaque sizes are presented as a percentage of WT. Data are mean ± SD and represent three independent experiments. One-way ANOVA with multiple comparisons to WT was used to calculate *P* value. ns, not significant, *P < 0.05; ***P < 0.001. (**C**) CD-1 mice were infected with each strain. Spleens and livers were collected 48 h post-infection for CFU quantification. Solid lines indicate the median, and the data represent two pooled biological repeats with 7–9 mice per strain. L.o.d, limit of detection. One-way ANOVA, multiple comparisons to either WT or WT Δ*gshF* as indicated, was used to calculate the *P* values. ns, not significant, **P < 0.01; ***P < 0.001; ****P < 0.0001.

Despite ΔP1 stressosome mutants expressing lower PrfA levels, it was still unclear why these mutants formed mixed-size plaques. We asked whether the mixed-size plaque phenotype observed in the ΔP1 stressosome mutants could be explained by the difference in PrfA activation within bacteria populations. We hypothesized that the glutathione availability either within bacteria or inside the host cells prior to infection may affect the outcome of the plaque formation, in line with previous reports that aimed to uncouple *prfA* transcription from PrfA abundance and activation (23, 28). To address this, we compared the virulence of the mutants in the presence of PrfA*, which is a constitutively active variant of PrfA that functions independently of its cofactor glutathione. Interestingly, ΔP1 *sigB::Tn* carrying PrfA* still displayed the mixed-size plaque phenotype that mirrored the virulence of the parent ΔP1 *sigB::Tn* strain (Fig. 4A). Similarly, deletion of the −10 sequence of the P2 promoter in a ΔP1 background (ΔP1P2) that phenocopies ΔP1 *sigB::Tn* mixed-size plaque was not rescued by PrfA*. Together, this suggests that enhanced PrfA activation prior to or during the infection is not sufficient to compensate for the absence of functional P1 and stressosome-dependent P2 promoters.

Next, we examined the relative contribution of P1 and P2 promoters individually during infection in the absence of functional P3 promoter. Since deleting P3 disrupts the expression of the bicistronic *plcA-prfA* transcript, we examined the role of P1 and P2 promoters in *Lm* lacking glutathione synthase (GshF), the only listerial enzyme that produces glutathione. In this background, we expected limited activity from the P3 promoter *in vivo* because transcription from the P3 promoter relies on the binding of the glutathione-PrfA complex to the PrfA box upstream of P3 (29, 30). Therefore, any changes in Δ*gshF Lm* virulence will reflect the activity of P1 or the SigmaB-dependent P2 promoter or both. A transposon insertion in *gshF* was transduced into *Lm* harboring *prfA* promoter deletions including ΔP1 (−10) or ΔP2 (−10) or ΔP1P2 (−10) (Fig. 4B). In the presence of functional GshF, the virulence of ΔP2 *Lm* was comparable to WT (Fig. 4B). Δ*gshF Lm* forms a small plaque, approximately 30% the size of WT, as shown previously (6). Interestingly, in a *gshF::Tn* background, deletion of either P1 and/or P2 resulted in no visible plaques (ΔP1 *gshF::Tn*, ΔP1P2 *gshF::Tn*) or barely detectable plaques (ΔP2 *gshF::Tn*), suggesting that when glutathione levels are low, both promoters are required to maintain virulence (Fig. 4B). Unlike ΔP1, deletion of P2 had no defect in mice, whereas deletion of both ΔP1 and P2 resulted in a 3-log defect in the spleen and almost complete clearance in the liver (Fig. 4C). ΔP1 *gshF::Tn* had a 2-log defect in the spleen and complete clearance in the liver, while ΔP2 *gshF::Tn* exhibited 0.5-log and 1-log attenuation defect in the spleen and liver respectively compared to WT Δ*gshF*. ΔP1P2 *gshF::Tn* led to near clearance in the spleen and the liver, suggesting that when glutathione production is low, *Lm* relies on both P1 and P2 promoters for pathogenesis *in vivo*.

### Deletion of the stressosome operon affects virulence *in vivo* in a ΔP1 background

**Fig 5:**
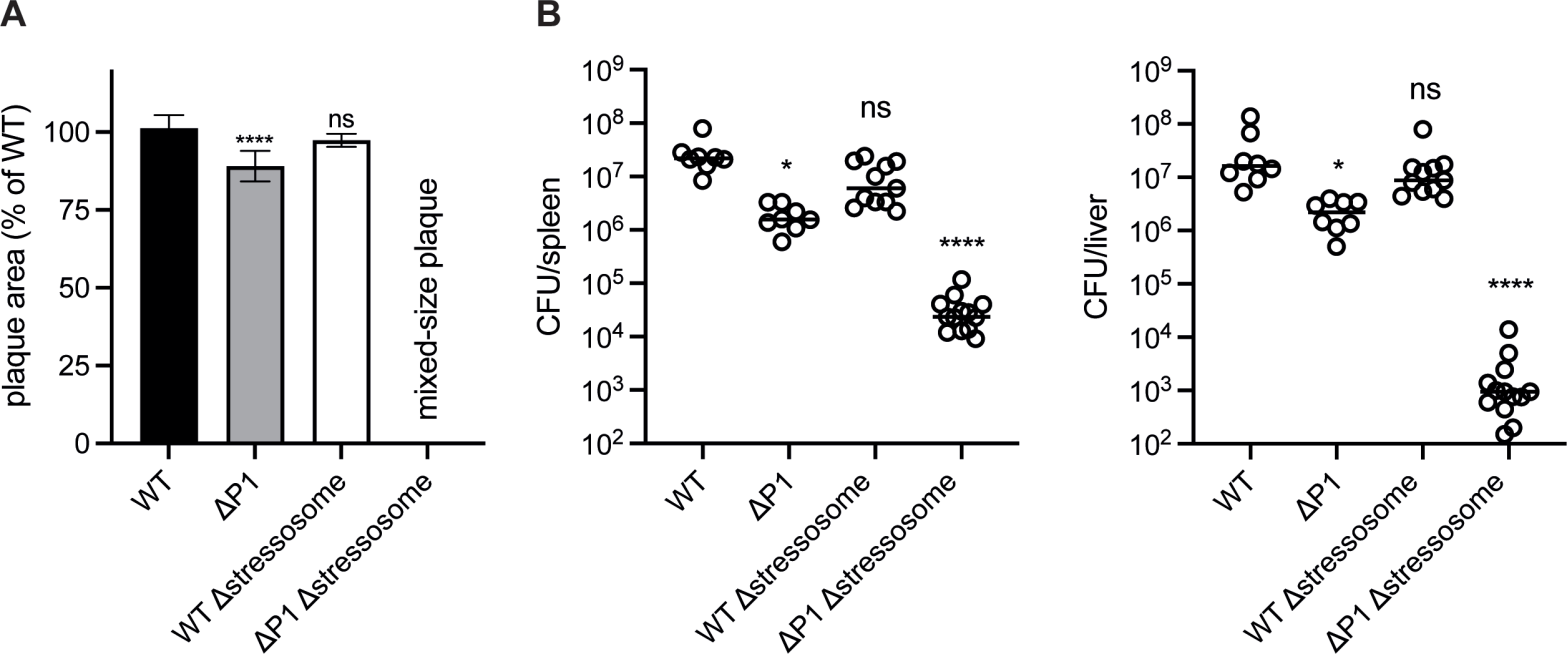
Analysis of the stressosome operon deletion mutants. (**A**) Plaque formation of WT and ΔP1 Δstressosome was measured in L2 cells and presented as percentage of WT. Data represent at least three independent experiments, and error bars indicate SD. *P* values were calculated using one-way ANOVA, multiple comparisons with WT. ns, not significant, ****P < 0.0001. (**B**) CD-1 mice were infected with WT and ΔP1 Δstressosome mutants, and CFUs were measured 48 h post-infection in spleens and livers. Solid lines indicate medians. Data represent two biological repeats combining 8–13 mice per strain. *P* values were calculated using one-way ANOVA, multiple comparisons with WT control strain. ns, not significant, *P < 0.05; ****P < 0.0001.

Having shown that individual stressosome signaling components play a role during intracellular infection, we next aimed to investigate how the deletion of the entire ~5 kb stressosome operon might affect bacterial virulence *in vivo*. We deleted all eight genes in the stressosome operon, including *rsbR1-rsbS-rsbT-rsbU-rsbV-rsbW-sigB-rsbX,* to generate WT/Δstressosome or ΔP1/Δstressosome strains. ΔP1/Δstressosome formed mixed-size plaque (Fig. 5A) similar to individual stressosome component mutants in a ΔP1 background. We were unable to generate a strain carrying all eight genes at an ectopic locus using the P*_hyper_* promoter in either WT or WT/ΔP1/Δstressosome backgrounds, suggesting that constitutive expression of the stressosome is detrimental for *Lm*. We constructed strains harboring all eight genes under the control of the P*_native_* promoter; however, this did not restore the phenotype, implying that the stressosome might be differently regulated at the transcriptional level (Fig. S3A). The virulence in mice of *Lm* lacking the entire stressosome operon was comparable to WT *Lm* (Fig. 5B). The ΔP1 parent strain was mildly impaired in both the liver and the spleen, consistent with the minor defect observed in the plaque assay (Fig. 5A). However, in a ΔP1 background, the strain lacking all stressosome components exhibited a 2-log defect in the spleen and a 4-log defect in the liver compared to ΔP1. This result highlighted that the stressosome is functional and able to respond to stress signals by activating the P2 promoter upstream of *prfA in vivo*.

### RsbR1 is the only RsbR paralog involved in stress detection *in vivo*

**Fig 6:**
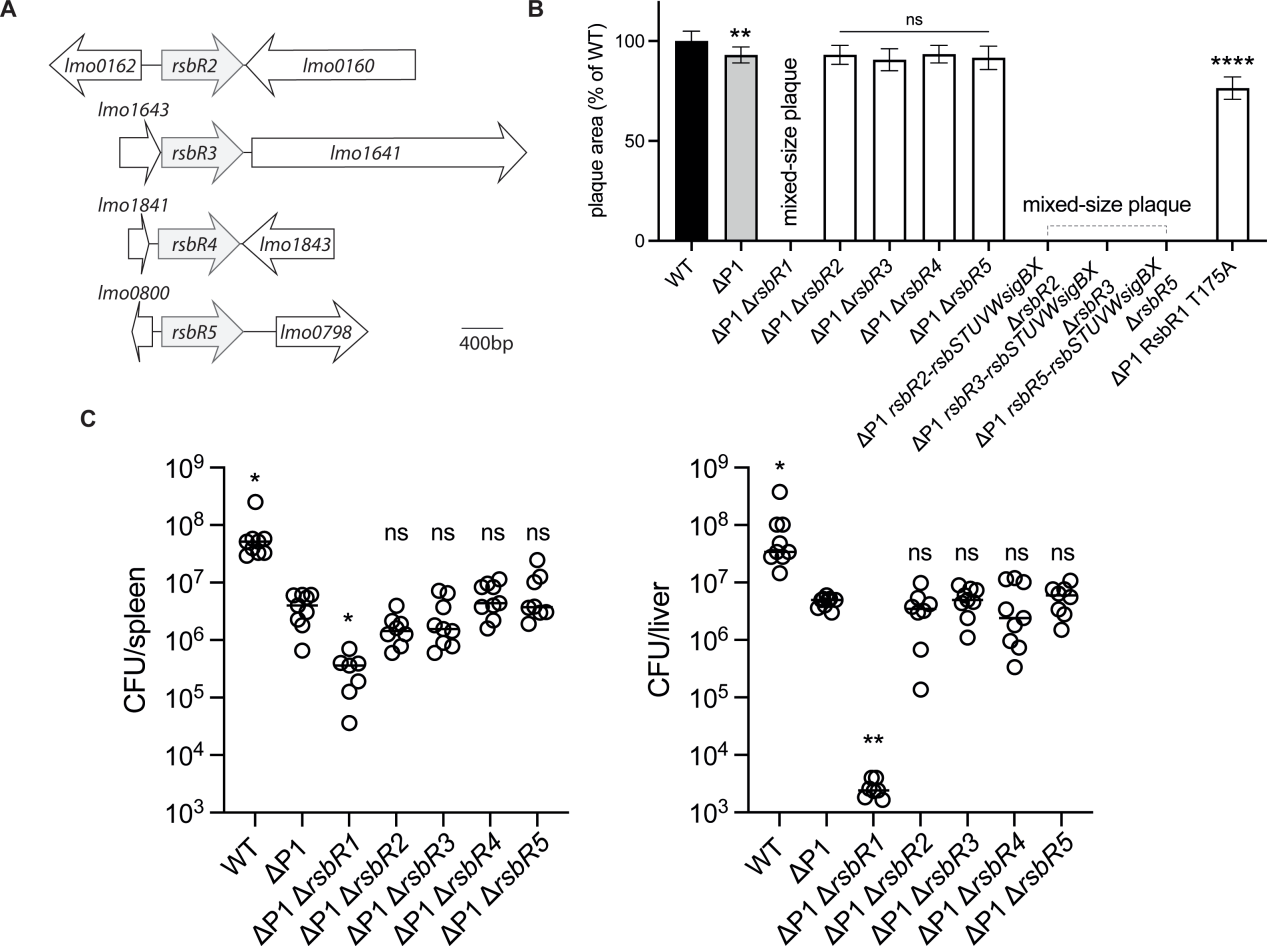
Characterization of RsbR paralog mutants *in vivo*. (**A**) Schematic representation of *rsbR*2*–rsbR*5 paralog loci in *Lm*. (**B**) Plaque assay of RsbR paralog and RsbR1 mutants in ΔP1 background. Plaque area is shown as a percentage of WT. Error bars indicate SD and one-way ANOVA, multiple comparisons with WT, was used to calculate *P* values. ns, not significant, **P < 0.01. (**C**) Virulence of RsbR paralog mutants in mice is presented as CFUs per mouse in spleens and livers after 48 h. Two biological repeats of 7–9 mice per strain. Solid lines represent the median. *P* values were calculated using one-way ANOVA, multiple comparisons with ΔP1. ns, not significant, *P < 0.05; **P < 0.01.

RsbR and RsbS form the core of the stressosome complex. There are five predicted RsbR paralogs in *Lm* (Fig. 6A), and each of the paralogs shares the STAT domain that interacts with RsbS and a variable domain thought to be involved in signal sensing (31). Previously, it was shown that stressosomes copurify with different RsbR paralogs *in vitro* (31). Each of the stressosome paralogs is predicted to have a variable sensory domain, implying that each RsbR paralog may respond to a specific stress signal (32, 33). Given that the disruption of the stressosome leads to a virulence defect in ΔP1, we assessed whether any of the RsbR paralogs were involved in stress sensing in a ΔP1 strain during infection. Deletion of the paralogs in the WT background had no plaque defect (Fig. S3B). Out of five RsbR mutants, only ΔP1/Δ*rsbR1* showed a mixed-size plaque phenotype mirroring the plaque defect displayed by ΔP1/Δstressosome (Fig. 6B). Our attempts to complement ΔP1/Δ*rsbR1* were unsuccessful. Since *rsbR1* is the first gene in the stressosome operon, it is possible that the defect observed with ΔP1 Δ*rsbR1* is due to disrupted expression of the remaining stressosome genes in the operon. To address this, we substituted the ORF of *rsbR1* with each individual *rsbR* paralog ORF (Fig. 6B). We were unable to construct *Lm* harboring *rsbR4* in place of *rsbR1* in the stressosome locus, suggesting that it might be toxic. *Lm* encoding either *rsbR2*, *rsbR3,* or *rsbR5* in place of the *rsbR1* ORF displayed a mixed plaque phenotype similar to ΔP1/Δ*rsbR1*. Additionally, ΔP1 RsbR1 T175A harboring a point mutation in the residue that has previously been shown to be important for stressosome activation (34) was attenuated in L2 plaque assay (Fig. 6B). Unlike Δ*rsbR1*, RsbR T175A still forms the stressosome; however, it resembles the inactive stressosome state (Allison Williams unpublished data), which can explain the difference between ΔP1/Δ*rsbR1* and ΔP1 RsbR T175A phenotypes. *In vivo* analysis of ΔP1 paralog mutants showed that only ΔP1/Δ*rsbR1* was severely attenuated, consistent with the plaque phenotype (Fig. 6C).

## DISCUSSION

PrfA is the master virulence regulator in *Lm,* and its expression is essential for bacterial survival during infection. In this study, we aimed to characterize the role of Sigma B-dependent regulation of *prfA* using a *Lm* strain (ΔP1) that contains only the Sigma B-dependent promoter directly upstream of *prfA* (Fig. 1A). The results of this study revealed a role for stressosomes in the regulation of *prfA* during infection.

WT *Lm* causes plaques in monolayers of fibroblasts that are remarkably homogeneous. In contrast, ΔP1 *Lm* stressosome mutants displayed a mixed-size plaque phenotype, which is unusual as it reflects that within genetically identical *Lm*, there may be different bacterial subpopulations each displaying variable virulence patterns (Fig. 1B). This defect is likely due to some bacteria displaying a slower rate of infection. Moreover, this slow rate of infection is transient as bacteria collected from either small or big plaques maintain mixed-size plaque phenotype upon reinfection. The heterogeneity can in part be explained by the changes in the PrfA expression levels that we see in ΔP1 *sigB::Tn* (Fig. 3). However, why within ΔP1 *sigB::Tn* some bacteria are fully virulent and form WT plaques albeit lower PrfA levels remains puzzling. The mixed-size plaque phenotype is likely driven by the host stress signals and is not dependent on glutathione activation of PrfA prior or during infection since PrfA* ΔP1 stressosome mutants similarly showed mixed-size plaque phenotype (Fig. 4A). It is possible that in addition to changes in PrfA expression levels, ΔP1 stressosome mutants undergo host-driven stochastic changes in post-transcriptional regulation of PrfA in the L2 fibroblast infection model. Indeed, stochastic expression of both PrfA and Sigma B has previously been reported at the single-cell level in *Lm* and *Bacillus subtilis* when exposed to different stress conditions *in vitro* (20, 35). Furthermore, one of the transposon insertions identified in our ΔP1 screen that, similarly to ΔP1 stressosome mutants, resulted in mixed-size plaques was the aminopeptidase Lmo1354, which has previously been shown to metabolically regulate PrfA at the posttranslational level (36). Although, due to technical limitations, we are unable to study the regulation of PrfA in each individual bacterium in a given plaque, it is clear that disrupted stressosome signaling leads to the loss of bacterial homogeneity, and the ΔP1 model provides an important foundation for understanding stressosome signaling in virulence.

Deletion of P1 and P2 promoters individually had a minor virulence defect *in vivo* (Fig. 4C). However, the P1/P2 promoter redundancy *in vivo* was abolished in *Lm gshF* mutants that were unable to synthesize glutathione (Fig. 4C). We conclude that when glutathione availability is limiting, Sigma A-dependent P1 and Sigma B-dependent P2 promoters are essential to maintain virulence. In addition to activating PrfA, glutathione is necessary to prevent redox-induced damage (37). Interestingly, *gshF* is part of the Sigma B-dependent regulon (12). Stressosomes might sense a glutathione-limited environment, such as that experienced in phagosomes, to first trigger *prfA* expression from the P2 promoter early during infection and then initiate *gshF*-dependent glutathione synthesis for PrfA activation during the subsequent stages of *Lm* pathogenesis.

Despite the numerous reports characterising the Sigma B response to different stresses *in vitro* including H_2_O_2_, temperature, ethanol, osmotic, and acid stress (38–42), it is unclear what signals activate the stressosomes *in vivo*. There are five RsbR paralogs in *Lm* proposed to be involved in stress sensing (43, 44). Among defined stress signals, blue light stress activates RsbR5 in *Lm*; however, the physiological role of this activation remains unknown (45–47). Our data demonstrate that RsbR1 is the only paralog that is able to respond to stress stimuli *in vivo* in a ΔP1 background (Fig. 6). We show that in ΔP1, stressosomes are important during both early stages of macrophage infection (Fig. 2), implying that the stressosome can respond to different stresses encountered during infection. Possible stressors may include redox regulation (Fig. 2F) and metabolic remodelling during infection (23, 48, 49). For example, in *Lm*, glycerol metabolism has been linked with virulence (50). Indeed, our ΔP1 mixed-size plaque screen also identified *lmo2456* and *lmo1652*, which are associated with sugar transport and metabolism, suggesting a possible crosstalk between metabolic regulation and stressosome signaling (Table 1).

Membrane stress has also been proposed as a direct stressosome activation signal (51, 52). In *Lm*, a small protein Prli42 was shown to anchor stressosomes to the membrane and activate the signaling cascade in response to oxidative stress (53). However, a recent study suggested that Prli42 is not required for RsbR1 association with *Lm* membranes (54). Our screen in ΔP1 *Lm* identified other candidates that could be involved in membrane stress sensing (Table 1), for example, *cydA* and *cydD*, predicted to encode components of the cytochrome bd respiratory oxidase, one of the two terminal oxidases found in *Lm* (55). Cytochrome D has been copurified with Prli42 along with the core stressosome components (53), suggesting a possible link between membrane stress sensing and stressosome signaling.

Considering that Sigma B regulates more than 300 genes including *prfA* and that the stressosome machinery is the second-largest signaling module found in bacteria, it is surprising and puzzling that deletion of the entire stressosome operon (or *sigB* alone) does not impact bacterial virulence *in vivo* in a WT background (Fig. 5), yet there are several possible explanations. First, stressosomes might play a role in adapting to a range of different environments that *Lm* encounters before entering the host (56). In the environmental bacterium *B. subtilis*, stressosomes respond to a number of stresses including temperature, acid, salt, oxidative, ethanol, and starvation (44, 57). Second, PrfA is essential for virulence, and the presence of multiple promoters upstream of *prfA* suggests that several complementary regulatory pathways exist to ensure the expression and activation of PrfA. Finally, the intravenous murine model of *Lm* infection may lack Sigma B-dependent stress signals that would reveal the importance of the stressosomes during listeriosis . For example, Sigma B is required for full virulence during oral infection in guinea pigs (58). The ΔP1 strain allowed us to reveal the robustness of the bacterial population in response to *in vivo* stresses in our cell-based and murine models of infection. We propose that in *Lm,* stressosomes play a role in activating virulence by controlling bacterial heterogeneity in order to survive and spread within the host. The ΔP1 *Lm* strain provides a platform to dissect further how various Sigma B-dependent signals are integrated to promote pathogenesis of *Lm in vivo*, which will expand our understanding of how intracellular pathogens regulate their virulence in response to stress.

## MATERIALS AND METHODS

### Bacterial culture strains and genetic manipulations

*E. coli* and *Lm* strains used in the study are listed in Tables S1 and S2.

Gene deletions in *Lm* were performed by allelic exchange using either pKSV7-oriT (59) or pLIM1 (gift from Arne Rietsch) (60). Complementation was performed using integration plasmid pPL2 (61). All mutants were confirmed by PCR and sequencing. Primers used in the study are listed in Table S3.

Growth curve assays were performed in BHI broth (BD Biosciences) at 37°C in Tecan Spark plate reader. A detailed protocol for the growth assays is described in supplemental methods.

### Plaque assay

Plaque assays were performed as previously described (62). L2 fibroblast cells were seeded in six-well plates (Gibco) with 1.2 × 10^6^ cells per well and incubated overnight at 37°C prior to infection. *Lm* was grown overnight, and an MOI of 0.1 was used for infection. The plate was incubated at 37°C for 72 h and the plaques were scanned and quantified using ImageJ software (63). The experiments were performed at least three times for each strain.

### Transposon library generation and screening in L2 cells

ΔP1 *Lm* was electroporated with pJZ037 carrying the *himar1* transposon as previously described (64). Transposon insertions were sequenced using arbitrary PCR (65) and mapped to the 10403S genome (NCBI ref: NC_017544) and EGD-e (NCBI ref: NC_003210).

Screening was performed by culturing ΔP1 *Lm* transposon library stock to OD_600_ = 1 prior to infecting 4 × 10^6^ L2 cells in 10 mL of L2 media. Small plaques were recovered on BHI agar, and single colonies were used for subsequent rounds of L2 infection. Transduction of transposons into WT or ΔP1 backgrounds was performed using U153 phage as described previously (66). The transposon loci were confirmed by sequencing.

### Macrophage growth curve

BMMs were collected from 8-week-old female C57BL/6J mice (The Jackson Laboratory) and differentiated and cultured in bone marrow macrophage (BMM) media as described previously (67). Macrophages were plated at 3 × 10^6^ per 60 mm non-tissue culture treated dishes (MIDSCI) containing fourteen 12 mm coverslips (ThermoFisher) in BMM media supplemented with 100 ug/mL PAM3CSK4 overnight at 37°C. *Lm* was grown overnight at 30°C stationary and used at an MOI of 0.25 to infect macrophages, an MOI that results in approximately 1 bacterium/20 macrophages, and BMM infection was performed as previously described (68). The experiment was performed three times.

### Mouse infections

*Lm* was grown overnight at 30°C and then used to inoculate 3 mL of fresh BHI media and grown for 4 h at 37°C shaking until OD_600_ = 0.6–1. Eight-week-old CD-1 mice (Charles River) were infected intravenously with 1 × 10^5^ CFU per 200 µL in PBS. Forty-eight hours post-infection, mice were subject to euthanasia, and spleens and livers were harvested in 3–10 mL of 0.1% IGEPAL (Sigma) in water. The organs were homogenized and plated for CFUs on LB-strep agar. Graphs represent pooled data from at least two independent infections of three mice or greater for each *Lm* strain.

### Disk diffusion assays

*Lm* cultures were grown overnight to stationary phase at 30°C and 10^7^ bacteria were immobilized in 3 mL top agar (55°C, 0.8% NaCl and 0.8% bacto-agar) and spread over BHI plates. Sterile filter paper disks were soaked in 5 µL of a 1 M diamide solution (Sigma) or 5% H_2_O_2_ (Fisher) and placed in the center of an agar plate. The plates were incubated overnight at 37°C, and the diameter of the zone of inhibition was measured.

### Phagosome escape assay

The assay was performed as previously described (22, 69). Briefly, 2 × 10^5^ BMM from C57BL/6J mice were grown overnight and pre-treated with cytochalasin D (250 ng/mL) in fresh BMM media 30 min prior to infection. MOI of 10 was used to infect BMMs, resulting in approximately 2 bacteria/cell. The detailed protocol for *Lm* and p62 staining is described in supplemental methods.

### Western blotting

Whole-cell extracts preparation followed by Western blotting procedure and imaging is described in supplemental methods. The membrane was incubated with anti-PrfA antibody (PA5-144446 ThermoFisher, 3:10,000) and anti-P60 (Adipogen, 1:5,000) followed by AlexaFluor-647 goat-anti rabbit (Life Technologies, 1:10,000) and goat anti-mouse IRDye 800CW (Licor 1:10,000), respectively, in Odyssey blocking buffer:PBS (1:1). The experiment was performed three times using cell extracts prepared from three independently grown bacterial cultures.

### qRT-PCR

The detailed protocol for RNA isolation and qRT-PCR is described in supplemental methods.

### Statistical tests

Statistical analysis was performed using GraphPad Prism (GraphPad Software, La Jolla, CA, USA). Statistical tests used for data analysis for each experiment are specified in the figure legends.

## SUPPLEMENTAL MATERIAL

Supplemental material mbio.00719-25-s0001.docx Figures S1-S3, Tables S1-S3, and supplemental methods.

ASM does not own the copyrights to Supplemental Material that may be linked to, or accessed through, an article. The authors have granted ASM a non-exclusive, world-wide license to publish the Supplemental Material files. Please contact the corresponding author directly for reuse.
