# Supplementary material for: Stress-dependent activation of the Listeria monocytogenes virulence program ensures bacterial resilience during infection
Source: mBio. 2025 Apr 30;16(6):e00719-25. doi: 10.1128/mbio.00719-25 (PMC12153296; doi:10.1128/mbio.00719-25)
Supplement: Supplemental material — Figures S1-S3, Tables S1-S3, and supplemental methods. [file mbio.00719-25-s0001.docx]

**Supplemental Material**

**Stress-dependent activation of the *Listeria monocytogenes* virulence program ensures bacterial resilience during infection.**

**Mariya Lobanovska, ^a^ Ying Feng,^a*^ Jonathan Zhang,^a**^ Allison H. Williams,^b,c^ Daniel A. Portnoy^a,d#^**

**SUPPLEMENTAL FIGURES AND FIGURE LEGENDS: SUPPL FIGURES 1- 3**

**SUPPLEMENTAL TABLES S1-S3**

**SUPPLEMENTAL METHODS**

**SUPPLEMENTAL FIGURES AND FIGURE LEGENDS**

**
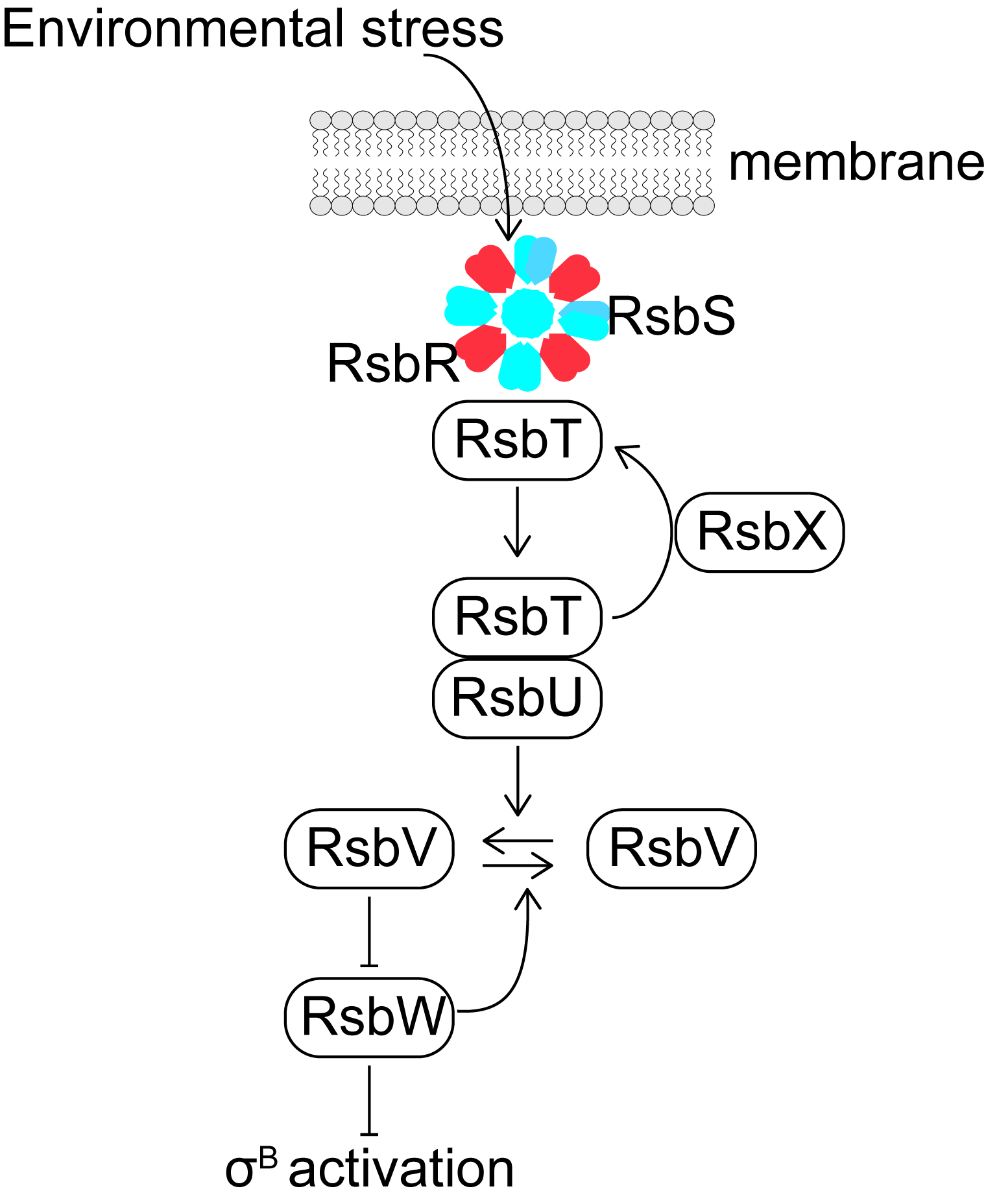
**

**Supplemental Figure 1** Stressosome signalling in *Lm*

Schematic diagram of the proposed stressosome signalling adapted from Williams *et al.* 2019. Briefly, the core of the stressosome is composed of RsbR and RsbS subunits. When the signal is detected by the core, RsbT kinase is activated and released from the stressosome core. RsbT binds the RsbU phosphatase that in turn dephosphorylates RsbV. Dephosphorylated RsbV binds RsbW, which is an anti-sigma factor that normally sequesters Sigma B. SigmaB becomes available to bind RNA polymerase and trigger Sigma B-dependent gene expression. Following a stress signal, the RsbX phosphatase is thought to dephosphorylate RsbR and RsbS thereby re-setting the stressosome to a non-stressed state.

**
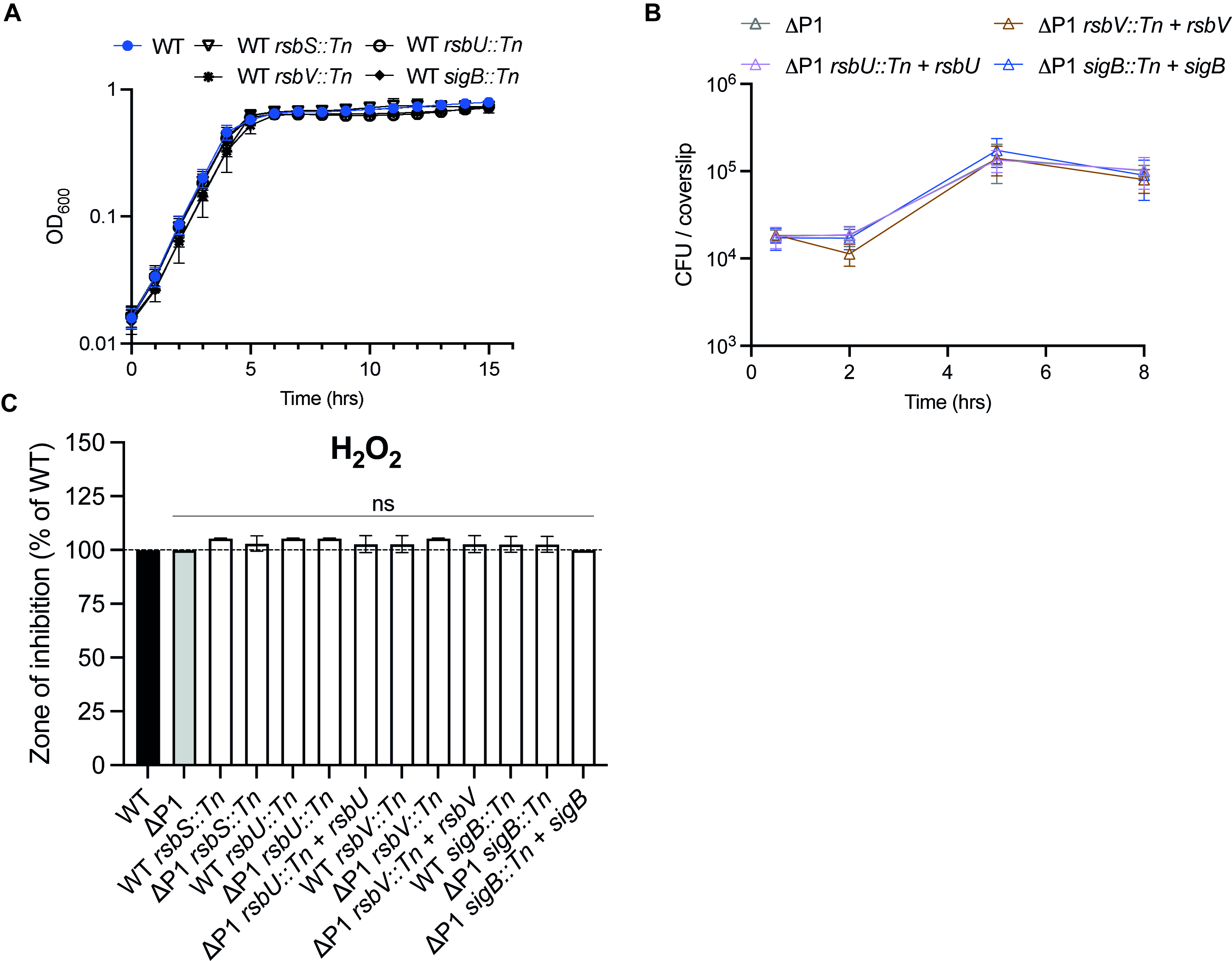
**

**Supplemental Figure 2** *In vitro* analysis of selected stressosome mutants

1. Growth of the indicated strains grown in BHI broth at 37^o^C with agitation over 15 hours. The graph represents pooled data from three independent biological repeats. Error bars indicate standard deviation. No statistical difference was detected between the strains.
2. Intracellular growth curves in bone marrow-derived macrophages were performed using the indicated strains. CFUs were measured at 0.5, 2, 5 and 8 hours post infection. The virulence of the ΔP1 stressosome transposon mutants with indicated genes under P*_hyper_* promoter is comparable to ΔP1 control. Data are combined from three biological repeats with error bars indicating SD.
3. Sensitivity of mutants to hydrogen peroxide (5% v/v) was measured using the diameter of the zone of inhibition and is presented as percentage of WT. Data are the mean and SD of three independent experiments. The stressosome transposon mutants did not show any sensitivity to H_2_O_2_. One-way ANOVA with multiple comparisons to WT was used to calculate the *p* values. ns, not significant.

**
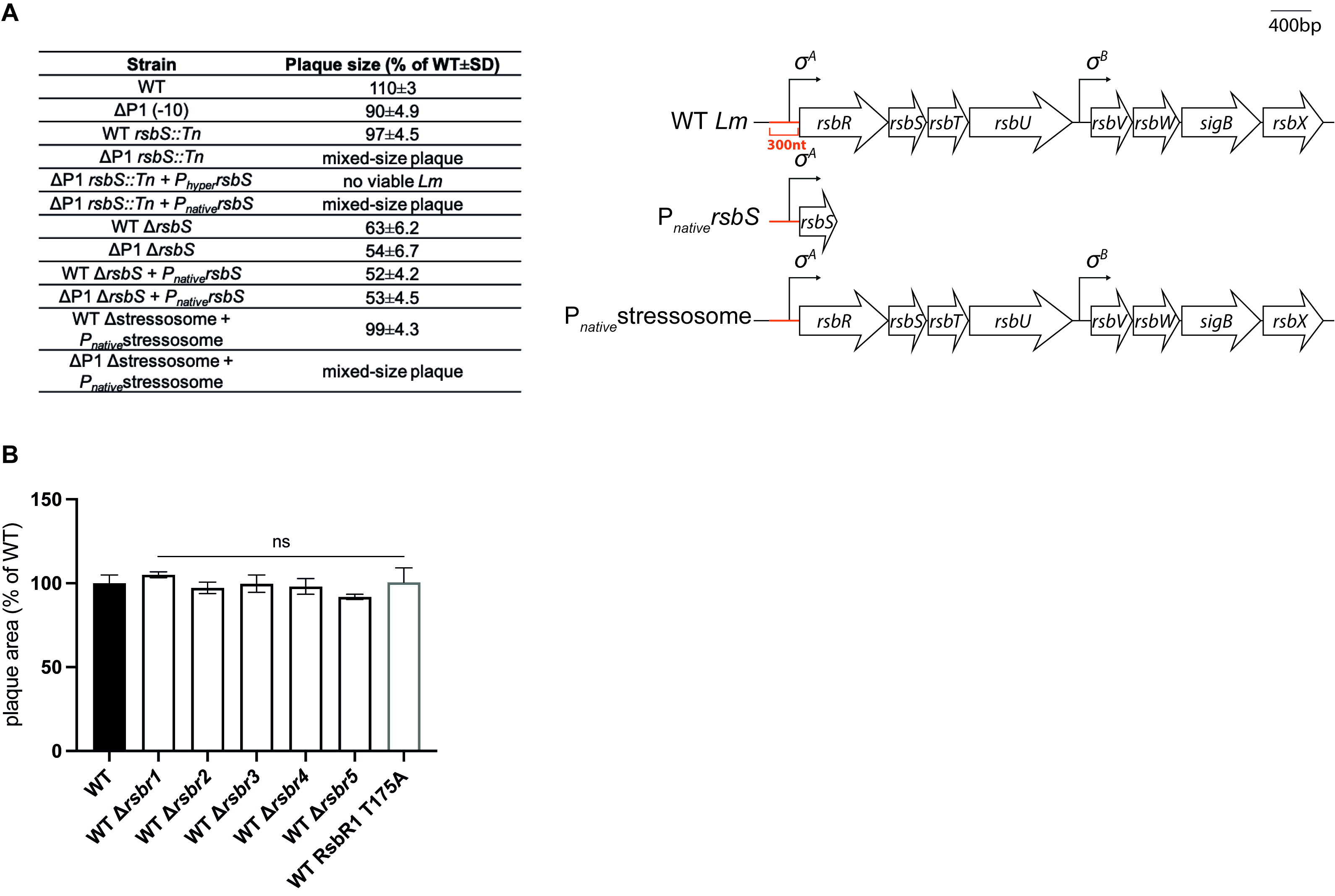
**

**Supplemental Figure 3** Characterization of selected stressosome mutants in L2 plaque assay

1. Plaque area of the indicated strains presented as percentage of WT. Complementation with P*_native_*-*rsbS*, which contains a 300 nt sequence upstream of *rsbR1* did not restore the mixed size plaque phenotype in ΔP1 *rsbS::Tn*, suggesting a more complex level of transcriptional regulation.

Deletion of *rsbS* results in plaque defect in both WT and ΔP1 backgrounds likely due to dysregulation of the downstream stressosome signalling components. Complementation with P*_native_*-*rsbS* constructs did not restore WT/ΔP1 Δ*rsbS* the plaque defect. Schematic diagram shows the region of the native promoter of the stressosome operon taken to construct P*_native_*-*rsbS,* P*_native_*-stressosome*.* Based on the homology to *B. subtilis*, the stressosome operon contains two promoters, a Sigma A-dependent promoter upstream of *rsbR1* and an internal Sigma B promoter upstream of *rsbV*. It is unclear how the first half of the operon encoding *rsbR-rsbS-rsbT-rsbU* is regulated at the transcriptional level. We were unable to complement WT/ΔP1Δstressosome mutants with the eight gene stressosome construct under P*_native_* promoter, suggesting that there might be additional regulatory elements beyond the 300 nucleotides upstream of *rsbR1* ORF involved in the expression of the stressosome operon genes.

1. Growth and spread of WT *rsbR* paralog mutants and WT T175A RsbR1 was measured using the L2 plaque assay. Plaque formation was measured 3 days post infection and plaque sizes are presented as percentage of WT. Data are mean ±SD of three independent experiments. One-way ANOVA with multiple comparisons to WT was used to calculate *p* value. ns, not significant.

**SUPPLEMENTAL TABLES S1-S1**

**Table S1** List of *E. coli* strains used in the study

| **Stock** | **Background** | **Plasmid** | **Reference** |
| --- | --- | --- | --- |
|  | *E. coli* SM10 |  | (1) |
| DP-E7549 | *E. coli* SM10 | pLIM | Gift from Arne Rietsch |
| DP-E7589 | *E. coli* SM10 | pKSV7.Δ*rsbS* | This study |
| DP-E7590 | *E. coli* SM10 | pPL2x.p*Nativ*e.*rsbS* | This study |
| DP-E7591 | *E. coli* SM10 | pPL2x.p*Hyper*.*rsbU* | This study |
| DP-E7592 | *E. coli* SM10 | pPL2x.p*Hyper*.*rsbV* | This study |
| DP-E7593 | *E. coli* SM10 | pPL2x.p*Hyper*.*sigB* | This study |
| DP-E7594 | *E. coli* SM10 | pLIM.Δstressosome | This study |
| DP-E7595 | *E. coli* SM10 | pPL2x.p*Native*.stressosome | This study |
| DP-E7596 | *E. coli* SM10 | pLIM.Δ*rsbR1* | This study |
| DP-E7597 | *E. coli* SM10 | pKSV7.*rsbR1*T175A | This study |
| DP-E7598 | *E. coli* SM10 | pLIM.Δ*rsbR2* | This study |
| DP-E7599 | *E. coli* SM10 | pKSV7.Δ*rsbR3* | This study |
| DP-E7600 | *E. coli* SM10 | pLIM.Δ*rsbR4* | This study |
| DP-E7601 | *E. coli* SM10 | pKSV7.Δ*rsbR5* | This study |
| DP-E7689 | *E. coli* SM10 | pPL2x.WT-*plcA*-*prfA* | This study |
| DP-E7690 | *E. coli* SM10 | pPL2x.*plcA*-ΔP1 (-10) *prfA* | This study |
| DP-E7691 | *E. coli* SM10 | pPL2x.*plcA*-ΔP1 (-10) *prfA** (G145S) | This study |
| DP-E7692 | *E. coli* SM10 | pPL2x.*plcA*-ΔP1P2 (-10) *prfA* | This study |
| DP-E7693 | *E. coli* SM10 | pPL2x.*plcA-*ΔP1P2 (-10) *prfA** (G145S) | This study |
| DP-E7694 | *E. coli* SM10 | pLIM. Δ*rsbR1+rsbR2* | This study |
| DP-E7695 | *E. coli* SM10 | pLIM. Δ*rsbR1+rsbR3* | This study |
| DP-E7696 | *E. coli* SM10 | pLIM. Δ*rsbR1+rsbR5* | This study |

**Table S2** List of *L. monocytogenes* used in the study

| **Stock** | **Background** | **Strain name** | **Reference** |
| --- | --- | --- | --- |
|  | *L. monocytogenes* 10403S | WT | (2) |
| DP-L1956 | *L. monocytogenes* 10403S | ΔP1 (-10) | (3) |
| DP-L7602 | *L. monocytogenes* 10403S | *rsbS::Tn* | This study |
| DP-L7603 | *L. monocytogenes* 10403S | *rsbU::Tn* | This study |
| DP-L7604 | *L. monocytogenes* 10403S | *rsbV::Tn* | This study |
| DP-L7605 | *L. monocytogenes* 10403S | *sigB::Tn* | This study |
| DP-L7606 | *L. monocytogenes* 10403S | *cydA::Tn* | This study |
| DP-L7607 | *L. monocytogenes* 10403S | *cydD::Tn* | This study |
| DP-L7608 | *L. monocytogenes* 10403S | *pgm::Tn* | This study |
| DP-L7609 | *L. monocytogenes* 10403S | *lmo1652::Tn* | This study |
| DP-L7610 | *L. monocytogenes* 10403S | ΔP1 *rsbS::Tn* | This study |
| DP-L7611 | *L. monocytogenes* 10403S | ΔP1 *rsbU::Tn* | This study |
| DP-L7612 | *L. monocytogenes* 10403S | ΔP1 *rsbV::Tn* | This study |
| DP-L7613 | *L. monocytogenes* 10403S | ΔP1 *sigB::Tn* | This study |
| DP-L7614 | *L. monocytogenes* 10403S | ΔP1 *cydA::Tn* | This study |
| DP-L7615 | *L. monocytogenes* 10403S | ΔP1 *cydD::Tn* | This study |
| DP-L7616 | *L. monocytogenes* 10403S | ΔP1 *pgm::Tn* | This study |
| DP-L7617 | *L. monocytogenes* 10403S | ΔP1 *lmo1652::Tn* | This study |
| DP-L7679 | *L. monocytogenes* 10403S | *WT lmo1354::Tn* | This study |
| DP-L7680 | *L. monocytogenes* 10403S | ΔP1 *lmo1354::Tn* | This study |
| DP-L7618 | *L. monocytogenes* 10403S | ΔP1 *rsbS::Tn + P_native_rsbS* | This study |
| DP-L7619 | *L. monocytogenes* 10403S | ΔP1 *rsbU::Tn + rsbU* | This study |
| DP-L7620 | *L. monocytogenes* 10403S | ΔP1 *rsbV::Tn + rsbV* | This study |
| DP-L7621 | *L. monocytogenes* 10403S | ΔP1 *sigB::Tn + sigB* | This study |
| DP-L7622 | *L. monocytogenes* 10403S | WT Δ*rsbS* | This study |
| DP-L7623 | *L. monocytogenes* 10403S | ΔP1 Δ*rsbS* | This study |
| DP-L7624 | *L. monocytogenes* 10403S | WT Δ*stressosome* | This study |
| DP-L7625 | *L. monocytogenes* 10403S | ΔP1 Δ*stressosome* | This study |
| DP-L7626 | *L. monocytogenes* 10403S | WT Δstressosome *+ P_native_*stressosome | This study |
| DP-L7627 | *L. monocytogenes* 10403S | ΔP1 Δstressosome *+ P_native_*stressosome | This study |
| DP-L7628 | *L. monocytogenes* 10403S | WT Δ*rsbR1* | This study |
| DP-L7629 | *L. monocytogenes* 10403S | ΔP1 Δ*rsbR1* | This study |
| DP-L7630 | *L. monocytogenes* 10403S | WT *rsbR1*T175A | This study |
| DP-L7631 | *L. monocytogenes* 10403S | ΔP1 *rsbR1*T175A | This study |
| DP-L7632 | *L. monocytogenes* 10403S | WT Δ*rsbR2* | This study |
| DP-L7633 | *L. monocytogenes* 10403S | ΔP1 Δ*rsbR2* | This study |
| DP-L7634 | *L. monocytogenes* 10403S | WT Δ*rsbR3* | This study |
| DP-L7635 | *L. monocytogenes* 10403S | ΔP1 Δ*rsbR3* | This study |
| DP-L7636 | *L. monocytogenes* 10403S | WT Δ*rsbR4* | This study |
| DP-L7637 | *L. monocytogenes* 10403S | ΔP1 Δ*rsbR4* | This study |
| DP-L7638 | *L. monocytogenes* 10403S | WT Δ*rsbR5* | This study |
| DP-L7639 | *L. monocytogenes* 10403S | ΔP1 Δ*rsbR5* | This study |
| DP-L6430 | *L. monocytogenes* 10403S | *gshF::Tn* | (4) |
| DP-L7640 | *L. monocytogenes* 10403S | ΔP1 (-10) *gshF::Tn* | This study |
| DP-L1957 | *L. monocytogenes* 10403S | ΔP2 (-10) | (3) |
| DP-L7641 | *L. monocytogenes* 10403S | ΔP2 (-10) *gshF::Tn* | This study |
| DP-L1964 | *L. monocytogenes* 10403S | ΔP1P2 (-10) | (3) |
| DP-L7642 | *L. monocytogenes* 10403S | ΔP1P2 (-10) *gshF::Tn* | This study |
| DP-L1865 | *L. monocytogenes* 10403S | ΔP1 (-35) | (3) |
| DP-L7643 | *L. monocytogenes* 10403S | ΔP1 (-35) *gshF::Tn* | This study |
| DP-L7681 | *L. monocytogenes* 10403S | *sigB::Tn* Δ*prfA* P3*plcA*- ΔP1 (-10) *prfA** (G145S) pPL2x | This study |
| DP-L7682 | *L. monocytogenes* 10403S | Δ*prfA* P3*plcA-* ΔP1 (-10) *prfA* pPL2x | This study |
| DP-L7683 | *L. monocytogenes* 10403S | Δ*prfA* P3*plcA*- ΔP1 (-10) *prfA** (G145S) pPL2x | This study |
| DP-L7684 | *L. monocytogenes* 10403S | Δ*prfA* P3*plcA*- ΔP1P2 (-10) *prfA* pPL2x | This study |
| DP-L7685 | *L. monocytogenes* 10403S | Δ*prfA* P3*plcA*- ΔP1P2 (-10) *prfA** (G145S) pPL2x | This study |
| DP-L7686 | *L. monocytogenes* 10403S | *rsbR2-rsbSTUVWsigBrsbX* | This study |
| DP-L7687 | *L. monocytogenes* 10403S | *rsbR3-rsbSTUVWsigBrsbX* | This study |
| DP-L7688 | *L. monocytogenes* 10403S | *rsbR5-rsbSTUVWsigBrsbX* | This study |
| DP-L4317 | *L. monocytogenes* 10403S | Δ*prfA* | (5) |

**Table S3** List of oligonucleotides used in the study

| **Target gene** | **Description** | **Forward primer 5' - 3'** | **Reverse primer 5' - 3'** |
| --- | --- | --- | --- |
| *ΔrsbS* | *upstream fragment* | aaagattttgcaaacttcatccg | aattatctttacacaggattggaatgtcatagtattcacccctctttttctactatc |
|  | *downstream fragment* | atgacattccaatcctgtgtaaag | tttttctagctctccttaccg |
| *P_native_rsbS* | *P_native_ promoter* | tcactaaagggaacaaaagctggtaccaaggtaaacaaggcacttgaag | cattcacctaactttaagattggtatccccaccagttattttccaacctttctccac |
|  | *rsbS* | ggggataccaatcttaaagttagg | tcattcccccaattcctgtttaag |
| *rsbU* | *rsbU ORF* | acatggaaaaagcttttgaggg | tacaaataagcgtaaagtcatcatg |
| *rsbV* | *rsbV ORF* | gtatagaaataaaagaacgtgatactgac | tgcattgttgccattcatttc |
| *sigB* | *sigB ORF* | atgccaaaagtatctcaacc | attactccacttcctcattctg |
| *Δstressosome* | *upstream fragment* | agaaaagggctcgcgaattaag | tttgcagttattttccaacctttctccac |
|  | *downstream fragment* | agtggagaaaggttggaaaataactgcaaaaacaccttttaaacgtttgc | gaaaaataaataagacaagcatacaaatac |
| *P_native_* | *P_native_* | tcactaaagggaacaaaagctggtaccaaggtaaacaaggcacttgaag | ttattccggaaatttcccaacc |
| *stressosome* | *stressosome operon* |  |  |
| *ΔrsbR1* | *upstream fragment* | acatgattacgaattcgagctcggtacccggcacgtggaagctactc | cacagtatcagttattttccaacctttctccac |
|  | *downstream fragment* | gagaaaggttggaaaataactgatactgtggggataccaatcttaaag | catgcctgcaggtcgactctagaggatcccgttcattcccccaattcctg |
| *ΔrsbR2* | *upstream fragment* | aggaaacagctatgacatgattacgaattcgcaaaatttgcccgggatttg | taactacttttctttattctatgctc |
|  | *downstream fragment* | ttttgagcatagaataaagaaaagtagttattatacttagacttaaaggggttgtctggtgaaaaaccgtaagcgattg | acaactggttctgttgaattaac |
| *ΔrsbR3* | *upstream fragment* | ggaaccggactcaatcttg | atctgtactaaacttctccttttttcaccg |
|  | *downstream fragment* | cggtgaaaaaaggagaagtttagtacagatgaatggcactcg | gctacttcattcgcaatcac |
| *ΔrsbR4* | *upstream fragment* | aaacagctatgacatgattacgaattcaagaaacgtcaaaagctcatacg | gttgcaaatgcgataattagcagattttgttttg |
|  | *downstream fragment* | acaaaatctgctaattatcgcatttgcaaccttacgccaag | tggagaacttgcgaagcc |
| *ΔrsbR5* | *upstream fragment* | ttacctctatacttataatacatgtttaag | tgtttctccccctttgtaaatac |
|  | *downstream fragment* | taggaatgtatttacaaagggggagaaacacgcagaagatattgcaaaatagcccg | gccaagcaggagtattcggtag |
| *rsbR1 T175A* | *mutation at the residue T175A* | tttctatgattaacttggctctttctgcgtcaatcgttccaattaacggc | acgcagaaagagccaagttaatcatag |
| *ΔrsbR1+* | *upstream* | ACATGATTACGAATTCGAGCTCGGTACCCGGcacgtggaagctactc | cagttattttccaacctttctcc |
| *rsbR2* | *rsbR2* | taatagtggagaaaggttggaaaataactgatgaatgaatcgaatggaagtatg | aactttaagattggtatccccacagtatttatatagttctagtttgatcttgcaaaacagcc |
|  | *downstream* | atactgtggggataccaatc | CATGCCTGCAGGTCGACTCTAGAGGATCCCgttcattcccccaattcctg |
| *ΔrsbR1+* | *upstream* | ACATGATTACGAATTCGAGCTCGGTACCCGGcacgtggaagctactc | cagttattttccaacctttctcc |
| *rsbR3* | *rsbR3* | taatagtggagaaaggttggaaaataactgAtgcaaattaaggaatttttgattagtcg | ataaacattcacctaactttaagattggtatccccacagtatttaaacacctaaattggttaacgc |
|  | *downstream* | taccaatcttaaagttaggtgaatg | CATGCCTGCAGGTCGACTCTAGAGGATCCCgttcattcccccaattcctg |
| *ΔrsbR1+* | *upstream* | ACATGATTACGAATTCGAGCTCGGTACCCGGcacgtggaagctactc | cagttattttccaacctttctcc |
| *rsbR5* | *rsbR5* | taatagtggagaaaggttggaaaataactgAtgaccgcttatccacaattc | ttagtaaaattgtaatgcttctttgattg |
|  | *downstream* | acaatcaaagaagcattacaattttactaaatactgtggggataccaatc | CATGCCTGCAGGTCGACTCTAGAGGATCCCgttcattcccccaattcctg |
| *Pnative-plcA-prfA* |  | taacaaatgttaatgcctccac | ttaatttaattttccccaagtagcag |
| *prfA* (G145S)* |  | tatttgcAGTcaacttttaatcctgacctatgtg | taggtcaggattaaaagttgACTgcaaatagagccaagcttcc |

| cDNA synthesis for *prfA* | ccgtctagctctctctaatcgagccaagcttcccgttaatc |
| --- | --- |
| Strand-specific RT-PCR for *prfA* | ttgatacagaaacatcggttgg |
| cDNA synthesis for 16S | ccgtctagctctctctaatcgtgtgtagcccaggtcataag |
| Strand-specific RT-PCR for 16S | acccttgattttagttgccag |
| Strand-specific RT-PCR tag | ccgtctagctctctctaatcg |

**SUPPLEMENTAL METHODS**

**Phagosome escape assay staining and imagining**

Cells were incubated with the following antibodies: 1:1000 of rabbit anti-*Listeria* antiserum (Difco, 223021) and 1:200 of guinea pig anti-P62 (Fitzgerald, 20R-PP001) for 90 min followed by 1:2000 of rhodamine Red-X goat α-rabbit IgG, 1:2000 AlexaFluor-647 goat anti-guinea pig IgG (Invitrogen, A21450) for 1 hour. Cells were imaged with Keyence using 100X objective and the following channels: DAPI, Cy5, and red. Several frames were randomly selected and images from at least 3 independent experiments were collected using Keyence software and analysed using Keyence BZ software. A minimum 70 bacteria were scored for each strain per colocalization experiment.

**Western blotting**

Bacteria grown to mid-log (OD_600_=0.5-0.8) in iLSM+TCEP were used to prepare cell extracts. Bacterial culture volume was normalised to OD_600_=1 and then centrifuged at max speed. The pellet was washed once in PBS, centrifuged again and the pellets were resuspended in 100 μl of 1x LDS containing freshly added βME (2.5%). Bacterial suspensions were transferred to O-ring tubes containing 0.4g of zirconium beads and bead beating was performed for two rounds of 40 seconds at 6m/s in FastPrep-24 homogenizer (MPbio). Samples were centrifuged at max speed for 5 min at 4^o^C and the supernatant was transferred to a fresh tube. The samples were boiled for 5 min at 70^o^C prior to loading 20 μl on the 12% polyacrylamide gels. Following electrophoresis, the proteins were transferred to PVDF membrane using iBlot2 ThermoFisher (7min, 20V). The membranes were blocked overnight at 4^o^C in Odyssey blocking solution. Membranes were incubated with appropriate primary and secondary antibodies to detect PrfA and P60 for 1 hour at room temperature. Membranes were washed with TBS-0.1% Tween for 15 min 3 times following primary and secondary antibody incubations. The bands were visualized using LiCOR Odyssey cxl fluorescent imaging system. The images were used for integrated density measurements with ImageJ (6). The integrated density measurement for each band was normalized to the image background and then the ratio of the normalized integrated density between PrfA/P60 was calculated for each strain.

**RNA isolation and qRT-PCR analysis**

Bacterial strains were grown to mid-log (OD_600_=0.5-0.8) in iLSM+TCEP and 10 ml was used to isolate RNA. RNA isolation was adapted from (7, 8). Bacterial cultures were centrifuged at max speed and the pellet was resuspended in Disruption solution (10% glucose, 12.5 mM Tris-HCl pH 7.6, and fresh 5 mM EDTA). The suspension was transferred to O-ring tubes containing 0.4g of zirconium beads and 500 μl of acid-phenol:chloroform (pH=6 with IAA, 125:24:1). Bacteria were homogenized for 40s at 6m/s using FastPrep-24 bead beater (MPbio). The tubes were centrifuged and the supernatant was transferred to a fresh tube containing 100 μl of chloroform and 1ml of TRIzol (ThermoFisher). The samples were mixed and incubated at room temperature for 5 min prior to centrifugation for 5 min at max speed. The aqueous phase was subjected to two rounds of chloroform extraction and the aqueous phase was transferred to a fresh tube. RNA was precipitated for 2 hours at -20^o^C by addition of 70% ethanol. RNA was collected by centrifuging the samples for 20 min at 4^o^C. RNA pellet was washed with 100% ethanol and dissolved in 50 μl of DEPC-treated water. Following DNase treatment for 30 min at 37^o^C (Tubro DNase, ThermoFisher), RNA was extracted using phenol:chloroform. The aqueous phase was mixed with chloroform-isoamyl alcohol (24:1), mixed and centrifuged for 5 min at 4^o^C. The RNA was precipitated by addition of 1/10 of the volume of 3M Na acetate (pH 4.5) and ½ of the volume of 100% ethanol for 2 hours at -20^o^C. RNA pellets were collected by centrifugation at max speed at 4^o^C for 20 min. The pellets were washed with 70% ethanol and resuspended in 50 μl DEPC-treated water. RNA quality was examined on the agarose gel and quantified using nanodrop.

For qRT-PCR, 2.5 μg of RNA was used to synthesize cDNA using the primers listed in Table S3. Reverse transcription was performed using superscript III (Invitrogen) in a total volume of 20 μl. Samples were treated with RNase H for 20 min at 37^o^C and cDNA was purified using PCR purification kit and eluted in 50 μl nuclease free water. 2 μl of purified cDNA template was used in a total volume of 20 μl of KAPA SYBR qPCR master mix to quantify *prfA* and *16S* expression. Biorad CFX Maestro Software was used to collect qPCR data from CFX Opus Real-Time PCR system. The data was analyzed using ΔCt method (Ct is the threshold cycle) (9). *prfA* expression is presented as the normalized R value (2^^-Ct^) relative to 16S expression that was used as a control.

**REFERENCES FOR SUPPLEMENTAL MATERIAL**

1. Simon R, Priefer U, Pühler A. 1983. A Broad Host Range Mobilization System for In Vivo Genetic Engineering: Transposon Mutagenesis in Gram Negative Bacteria. Bio/Technology 1:784.

2. Bécavin C, Bouchier C, Lechat P, Archambaud C, Creno S, Gouin E, Wu Z, Kühbacher A, Brisse S, Pucciarelli MG, García-del Portillo F, Hain T, Portnoy DA, Chakraborty T, Lecuit M, Pizarro-Cerdá J, Moszer I, Bierne H, Cossart P. 2014. Comparison of widely used Listeria monocytogenes strains EGD, 10403S, and EGD-e highlights genomic variations underlying differences in pathogenicity. mBio 5:e00969-14.

3. Freitag NE, Portnoy DA. 1994. Dual promoters of the Listeria monocytogenes prfA transcriptional activator appear essential in vitro but are redundant in vivo. Molecular Microbiology 12:845-853.

4. Reniere ML, Whiteley AT, Hamilton KL, John SM, Lauer P, Brennan RG, Portnoy DA. 2015. Glutathione activates virulence gene expression of an intracellular pathogen. Nature 517:170-3.

5. Cheng LW, Portnoy DA. 2003. Drosophila S2 cells: an alternative infection model for Listeria monocytogenes. Cellular Microbiology 5:875-885.

6. Schneider CA, Rasband WS, Eliceiri KW. 2012. NIH Image to ImageJ: 25 years of image analysis. Nature Methods 9:671-675.

7. Ignatov D, Vaitkevicius K, Durand S, Cahoon L, Sandberg SS, Liu X, Kallipolitis BH, Rydén P, Freitag N, Condon C, Johansson J. 2020. An mRNA-mRNA Interaction Couples Expression of a Virulence Factor and Its Chaperone in Listeria monocytogenes. Cell Reports 30:4027-4040.e7.

8. Loh E, Dussurget O, Gripenland J, Vaitkevicius K, Tiensuu T, Mandin P, Repoila F, Buchrieser C, Cossart P, Johansson J. 2009. A trans-acting riboswitch controls expression of the virulence regulator PrfA in Listeria monocytogenes. Cell 139:770-9.

9. Schmittgen TD, Livak KJ. 2008. Analyzing real-time PCR data by the comparative CT method. Nature Protocols 3:1101-1108.
